# Lateral cutaneous nerve entrapment syndrome associated with appendicitis

**DOI:** 10.1093/jscr/rjag470

**Published:** 2026-06-18

**Authors:** Kazuma Kawahara, Yuki Kataoka, Takuya Miyagawa, Takashi Watari

**Affiliations:** Postgraduate Clinical Training Center, Min-iren Chuo Hospital, 2-1 Uzumasa Tsuchimoto-cho, Ukyo-ku, Kyoto-shi, Kyoto 616-8147, Japan; Center for Medical Education, Graduate School of Medicine, Nagoya University, 65 Tsurumai-cho, Showa-ku, Nagoya, Aichi 466-8550, Japan; Scientific Research Works Peer Support Group (SRWS-PSG), Osaka, Japan; Department of Internal Medicine, Kyoto Min-iren Asukai Hospital, 89 Tanaka Asukai-cho, Sakyo-ku, Kyoto-shi, Kyoto 606-8226, Japan; Department of Internal Medicine, Kyoto Min-iren Asukai Hospital, 89 Tanaka Asukai-cho, Sakyo-ku, Kyoto-shi, Kyoto 606-8226, Japan; Integrated Clinical Education Center, Kyoto University Hospital, 54 Shogoin Kawahara-cho, Sakyo-ku, Kyoto-shi, Kyoto 606-8507, Japan; General Medicine Center, Shimane University Hospital, 89-1 Enya-cho, Izumo-shi, Shimane 693-8501, Japan

**Keywords:** lateral cutaneous nerve entrapment syndrome, LACNES, trigger-point injection, appendicitis

## Abstract

A man in his 60s presented with localized right flank pain. Physical examination revealed a discrete tender point and a positive pinch sign. A trigger-point block led to immediate pain relief. Based on these findings, he was clinically diagnosed with lateral cutaneous nerve entrapment syndrome (LACNES). His Alvarado score was 7 and abdominal computed tomography revealed appendiceal swelling and surrounding fat stranding, indicating underlying appendicitis. The patient underwent emergency laparoscopic appendectomy. He made a full recovery with no recurrence of the pain. In this case, we speculate that appendicitis and localized peritonitis might have caused entrapment of the lateral cutaneous nerve as a result of muscle guarding. In patients with localized flank pain resembling LACNES, clinicians should not overlook the possibility of underlying intra-abdominal inflammatory conditions such as appendicitis.

## Introduction

Lateral cutaneous nerve entrapment syndrome (LACNES) and the related condition, anterior cutaneous nerve entrapment syndrome (ACNES), result from entrapment of intercostal nerve branches penetrating the abdominal fascia. LACNES typically presents with a localized dull pain or discomfort in the lateral abdomen. Precipitating factors include vertebral compression fractures, prior abdominal surgery, endoscopic procedures, and postural changes. Although these syndromes are increasingly recognized causes of abdominal wall pain, the underlying etiology is sometimes overlooked.

We report the case of a patient who presented with localized discomfort in the right flank and physical findings suggestive of LACNES in whom imaging and laboratory findings revealed concurrent appendicitis. Previous literature on LACNES has described mechanical and postural factors, procedures, and external stimuli as potential triggers, but has not considered intraperitoneal inflammation as a potential trigger. This case expands the clinical spectrum of LACNES by suggesting that intraperitoneal inflammation can act as a trigger, and has implications for the diagnostic approach to patients presenting with localized flank pain.

## Case presentation

A man in his 60s presented to our outpatient clinic with a 2-week history of right flank discomfort. He reported no radiation or aggravation with postural changes and no systemic symptoms such as fever, nausea, or weight loss. His medical history included hypertension, hyperuricemia, for which he was taking amlodipine and allopurinol, respectively.

On examination, he appeared well, and his vital signs were stable. The abdomen was soft with normal bowel sounds. A localized area of tenderness and sensory dullness (2–3 cm in diameter) was identified in the right flank at the level of T11 with a positive pinch sign (Video 1). Mild tenderness with muscle resistance was noted in the right lower quadrant, without generalized muscle guarding. McBurney’s and Lanz’s points were positive, whereas rebound tenderness, Rovsing’s sign, and the psoas sign were absent.

Laboratory tests showed leukocytosis (11 500 cells/μL, 75% neutrophils) and an elevated C-reactive protein level (10.1 mg/dL). The Alvarado score was 7, suggesting acute appendicitis. Abdominal computed tomography (CT) revealed an enlarged appendix with peri-appendiceal fat stranding, suggesting localized appendicitis (Fig. 1), but no vertebral abnormalities. The right external and internal oblique muscles were slightly thicker than those on the left (18.4 mm vs. 16.3 mm), with indistinct fascial margins, suggesting localized muscle tension.

**Figure 1:**
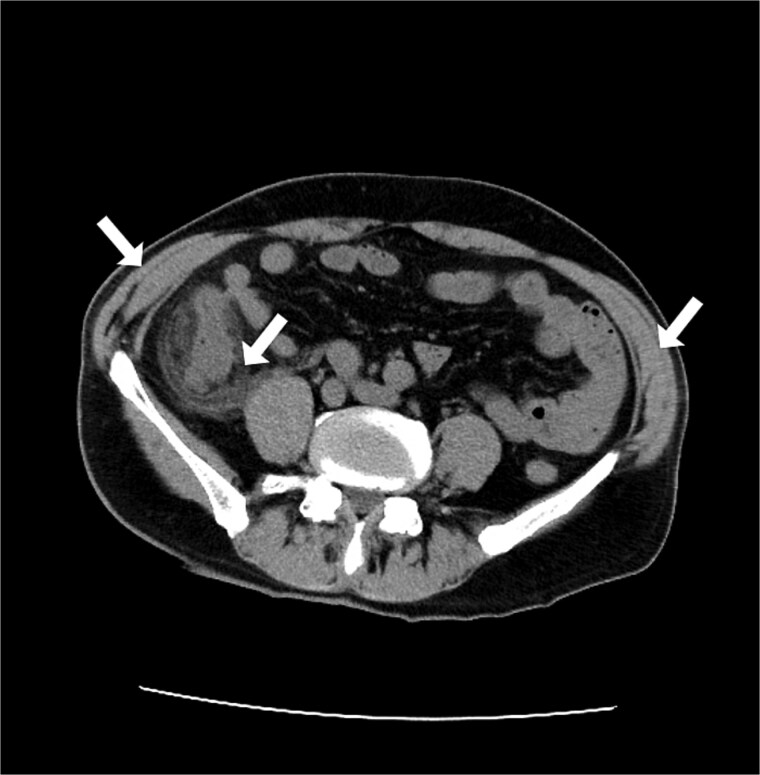
Plain abdominal CT showing an enlarged appendix with peri-appendiceal fat stranding, consistent with localized appendicitis. The right external and internal oblique muscles are slightly thicker than those on the left side, with indistinct fascial margins, suggesting localized muscle tension. No other potential causes of abdominal wall pain are apparent.

A trigger-point injection of 1% lidocaine was administered at the tender point (T11 level), resulting in immediate pain relief and the pinch sign became negative (Video 1).

Based on these findings, the patient was diagnosed with LACNES with concurrent appendicitis. He was referred to another hospital, where he underwent emergency laparoscopic appendectomy and was discharged on postoperative day 4. At the 2-week follow-up, the pain had completely resolved.

## Discussion

This patient had concurrent LACNES and appendicitis. Although causality cannot be established, we speculate that the LACNES might have been triggered by underlying subacute appendicitis. The differential diagnosis of localized flank pain includes ACNES, abdominal wall hernia, and radicular pain due to spinal disorders. LACNES was diagnosed based on the lateral localization of the pain, the presence of a positive pinch sign, and the immediate resolution of symptoms following a trigger-point injection, and imaging findings, and appendicitis was diagnosed based on the Alvarado score and imaging findings.

LACNES is characterized by entrapment of the lateral cutaneous branch of the intercostal nerve (T7–12) as it penetrates the abdominal fascia, resulting in localized abdominal wall pain and sensory disturbance [1, 2]. Awareness of LACNES as a cause of abdominal wall pain has increased in recent years. The median age of reported cases is approximately 50 years, with a female predominance [3]. The diagnosis of LACNES is based on satisfying at least three of the following four clinical criteria: (i) a history of localized flank pain lasting for at least 3 months, (ii) a fingertip-sized area of constant tenderness along the midaxillary line that produces sharp pain on compression, (iii) altered skin sensation (hypoesthesia, hyperesthesia, or temperature change) around the tender area, and (iv) a positive pinch sign outside the painful zone [1]. In the absence of a targeted physical examination, this condition often remains unrecognized, as imaging and laboratory test results are nonspecific and clinical ‘rule-in’ is inherently difficult [3]. In with LACNES the neuropathic pain is caused by entrapment or irritation of the cutaneous branches of the lower intercostal nerves. Because its pathophysiology involves localized nerve irritation, lidocaine injection into the tender point is the first-line treatment [1, 2], often providing immediate pain relief and serves as both diagnostic confirmation and treatment.

Mechanical and postural factors have been widely reported as triggers for LACNES and the related disorder, ACNES. Specifically, physiological changes such as pregnancy [4], surgical trauma such as open or laparoscopic surgery [5, 6], trauma such as T11 thoracic vertebral compression fractures [7], postural factors such as sudden changes in body position [8], and endoscopic examination [9, 10] have been reported as triggers. However, to our knowledge, LACNES linked to intraperitoneal inflammatory diseases such as appendicitis has not been reported previously.

Although a causal link between appendicitis and LACNES cannot be established, we speculate that in this case, localized peritonitis associated with appendicitis irritated the parietal peritoneum, causing reflex guarding of the right abdominal wall muscles, and that the resulting hypertrophy and tension led to entrapment of the lateral cutaneous branch of the intercostal nerve. The observation of muscle thickening and increased surrounding fat density in CT images supports this hypothesis, but it remains speculative and requires further investigation. To our knowledge, the possibility that localized abdominal wall muscle tension associated with intraperitoneal inflammation can trigger lateral cutaneous nerve entrapment, has not previously been proposed as a pathogenetic mechanism for LACNES. In patients with localized flank pain resembling LACNES, clinicians should not overlook the possibility of underlying intra-abdominal inflammatory conditions such as appendicitis.

## Supplementary Material

LACNES_VIDEO_rjag470 Video 1. Physical findings before and after trigger-point injection.
This video shows a localized tender point with associated sensory hyperalgesia and a positive pinch sign, supporting the diagnosis of lateral cutaneous nerve entrapment syndrome (LACNES). The pain resolves immediately following a trigger point block using 1% lidocaine.

## Data Availability

All relevant data are included in this report.
